# Efficacy of Adjunctive Bioactive Materials in the Treatment of Periodontal Intrabony Defects: A Systematic Review and Meta-Analysis

**DOI:** 10.1155/2018/8670832

**Published:** 2018-05-27

**Authors:** Shuai Zhou, Chengjia Sun, Shaohui Huang, Ximing Wu, Yan Zhao, Chunling Pan, Hongyan Wang, Junchao Liu, Qian Li, Yurong Kou

**Affiliations:** ^1^Department of Periodontology, School of Stomatology, China Medical University, Shenyang, Liaoning 110002, China; ^2^Department of Oral Biology, School of Stomatology, China Medical University, Shenyang, Liaoning 110002, China; ^3^Department of Oral and Maxillofacial Surgery, School of Stomatology, China Medical University, Shenyang, Liaoning 110002, China

## Abstract

**Objectives:**

Lots of bioactive materials have been additionally applied for the treatment of periodontal intrabony defect. However, there is dearth of studies to systematically evaluate the supplementary role of them in periodontal regeneration. The goal of this meta-analysis is to evaluate the adjunctive effects of bioactive materials such as platelet-rich plasma (PRP), platelet-rich fibrin (PRF), enamel matrix derivative (EMD), and amnion membrane (AM) on the outcomes of bone grafting treatment for periodontal intrabony defects.

**Methods:**

Articles published before December 2017 were searched electronically in three databases (PubMed, Embase, and Cochrane Central), with no date or language limits. Randomized controlled trials (RCTs) on the assessment of effectiveness of the four biomaterials in conjunction with demineralized freeze-dried bone allografts (DFDBA) in the treatment of periodontal intrabony defects were enrolled in this meta-analysis. Data were analyzed with STATA 12.

**Results:**

Nine studies were included. PRF and PRP significantly improved pocket depth (PD) reduction and clinical attachment loss (CAL) gain. Only PRF exhibited a positive result in recession reduction (RecRed). Only PRP showed a statistically significant increase in bone fill. AM merely gained more CAL. EMD did not improve any clinical outcome.

**Conclusion:**

Our data suggest that PRF/PRP could be taken as a preferred adjunct to facilitate periodontal regeneration of intrabony defects.

## 1. Introduction

Periodontitis, a main cause of tooth loss in adults, is characterized by the bacterially induced inflammation and breakdown of periodontal supporting tissue, which frequently results in the formation of intrabony defects. The ultimate goal of periodontal therapy is not only to arrest the periodontal disease progression but also to regenerate the original architecture and function of the periodontal complex, which involves the formation of new cementum on the tooth root, along with new periodontal attachment between newly formed bone and cementum [1]. The classic approach to periodontal regeneration to date is the use of filling materials to repair periodontal defects. Lots of techniques focusing on a quest for efficient defect filling materials have been developed, utilizing autografts, allografts, xenografts, and various man-made bone substitutes [2]. Autograft is considered the “gold standard” for periodontal graft material. But people resort more to other alternatives for periodontal repair in order to avoid a secondary surgical harvest site and surgical complications of ankylosis and root resorption. A variety of allografts, xenografts, and synthetic materials such as DFDBA, anorganic bovine bone, hydroxyapatite (HA), and tricalcium phosphate (TCP) are now available and widely used as regeneration materials. Also, a biocomposite poly(lactic-co-glycolic) acid/submicron size hydroxyapatite (PLGA/HA) was used successfully for periodontal regeneration [3]. These bone substitutes have been proven to be osteoconductive in repairing defects, serving as mechanical scaffolding for new bone formation. However, except for autogenous bone, the other grafting materials have no or questionable osteogenic and osteoinductive capacity, which restricted their regeneration efficiency. Hence, more effective regenerative approach is still the target to seek, notwithstanding the limited success gained.

Tissue engineering (TE) offers a promising strategy to facilitate reconstruction or regeneration of a particular tissue [4]. Seed cells, scaffold materials, and biological factors of microenvironment are the three essential elements of TE. The use of biological mediators may selectively enhance cellular repopulation of the periodontal wound. So a combination of biologically active agents with scaffold materials may positively influence the treatment of a periodontal intrabony defect by promoting robust periodontal regeneration [5]. Results from a recent series of studies anticipated that the combination may yield synergistic effects on correcting the periodontal defects where the graft materials may act as an osteoconductive scaffold maintaining the defect space, while the bioactive materials may induce formation of root cementum, periodontal ligament, and bone, mimicking the natural processes. EMD, PRP, PRF, and AM, the biomaterials processed from human bodies or animals, have been intensively investigated and clinically applied in periodontal regeneration in recent couple of decades [6–12]. EMD, made from developing porcine teeth, has been reported to contain a mixture of low molecular weight proteins, which may be absorbed into the hydroxyapatite and collagen fibers of the root surface and cause cementum formation [6–8]. PRP and PRF are the two generations of platelet concentrates (PCs), respectively, obtained after processing autologous whole blood samples, mostly through centrifugation. The preparation of PRP (the first PCs generation) requires anticoagulants at the moment of blood collection; bovine thrombin and calcium chloride have to be added when used in the gel form. In contrast, PRF (the second PCs generation), much more simply prepared, is nothing more than centrifuged blood without any additives [9]. Both PRP and PRF contain highly concentrated growth factors such as transforming growth factor-*β* (TGF-*β*), platelet derived growth factor (PDGF), vascular endothelial growth factor (VEGF), insulin-like growth factor (IGF), epithelial growth factor (EGF), and fibroblast growth factor-*β* (FGF-*β*), with platelets as the rich source, having potential to enhance wound healing and periodontal regeneration through modulating neoangiogenesis, cell proliferation, migration, differentiation, and other cellular functions [13]. Studies have indicated that TGF-*β*1 and VEGF-A worked together and could promote osteoblastic differentiation of bone marrow stromal cells in both cell culture and an animal model. Moreover, VEGF could strengthen BMP2-induced bone formation through regulation of angiogenesis [14, 15]. Allogenic AM, derived from the human placenta, is a thin, tough, transparent, absorbable composite membrane, made up of three major layers: a single epithelial layer, a thick basement membrane, and an avascular mesenchyme of collagen [10]. Owing to the plenty of growth factors trapped by the AM matrix, it has been evidenced to induce angiogenesis, facilitate cell migration, recruit mesenchymal progenitor cells, and exhibit anti-inflammatory, antimicrobial, and immunomodulatory properties, which helps in accelerating wound healing and tissue formation [11]. Besides, the lack of immunogenicity and relative ease of processing and procurement of AM also contribute to its wide application in tissue regeneration such as the reconstruction of skin, cornea, and conjunctiva [16–19]. Its use in periodontal defect restoration has just begun recently and has gained some positive outcomes [20–23].

Despite the fact that literature is replete with articles assessing the effects of bioactive materials on periodontal surgical treatment, results are often inconsistent and it is difficult to compare the clinical efficacy of them due to the varied forms of combination of bioactive materials and scaffold materials. To the best of our knowledge, there is dearth of studies to systematically evaluate the supplementary role of bioactive materials in periodontal defect regeneration. Which one is truly effective? Which one is better? The aim of the present systematic review and meta-analysis was to evaluate and compare the clinical outcomes of EMD, PRP, PRF, and AM in conjunction with DFDBA in patients with periodontal intrabony defects, which might have some guiding significance on clinical management strategy for the option of additional bioactive materials.

## 2. Methods

### 2.1. Search Protocol

We used ((demineralized freeze-dried bone allograft) OR (decalcified freeze-dried bone allograft) OR (DFDBA)) AND ((intrabony defect) OR (infrabony defect) OR (osseous defect)) as search terms. Electronic searches were conducted in PubMed, Embase, and Cochrane Central for scientific papers that were published until 22 December 2017 without regard to date or language restriction. We also evaluated studies that were cited in the reference lists of the included papers in case of missing relevant articles.

### 2.2. Studies Selection

This systematic review and meta-analysis included RCTs that compared the performances of DFDBA with or without one of the four bioactive materials (EMD, PRP, PRF, and AM) in patients with periodontal intrabony defects, with follow-up periods of >=6 months. The exclusion criteria included retrospective cohort studies, animal studies, in vitro studies, case reports, case series, and reviews.

### 2.3. Data Extraction and Quality Analysis

Basic information extracted from all included studies includes authors, publication year, volume and issue, study design, follow-up, number of participants, mean difference (MD) in PD reduction, CAL gain, RecRed, bone fill, and bone resorption between baseline and ultimate follow-up. Two authors (Shuai Zhou and Chengjia Sun) independently checked all the data from the included studies. Disagreements were solved by discussion or consultation with another author (Yurong Kou) who thereby helped to reach a final agreement.

According to Cochrane Handbook (available at: http://training.cochrane.org/handbooks), five main criteria were examined: random sequence generation (adequate, inadequate, and unclear), allocation concealment (adequate, inadequate, and unclear), blinding of outcome assessment (yes, no, and unclear), incomplete outcome data (yes, no, and unclear), and selective reporting (yes, no, and unclear). The studies were grouped into three categories after quality assessment: low risk of bias if all the criteria were met, moderate risk of bias if three or four criteria were met, and high risk of bias if <3 criteria were met.

### 2.4. Statistical Analysis

The clinical parameters were pooled from the included studies for meta-analysis. For one study, in which the mean ± SDs were unavailable, medians were treated as mean values directly and IQRs were used to estimate the SDs using the following formula: SD = IQR/1.35 [24, 25]. The authors worked out the mean difference (MD) and 95% confidence intervals (CI) using STATA 12 (StataCorp LP, College Station, TX, USA). The *I*^2^ test was used to quantify the effect of heterogeneity. Values up to 25% were classified as low heterogeneity, and values up to 50% or 70% were classified as medium or high heterogeneity, respectively. If the *I*^2^ test < 50%, the fixed-effects model was used; if there was significant heterogeneity among the included studies (*I*^2^ test > 50%), the random-effects model was employed. Subgroup analysis and sensitivity analysis were conducted to assess statistical stability. Begg's test and Egger's linear regression were used for evaluating publication bias.

## 3. Results

### 3.1. The Characteristics and Quality Evaluation of Included Articles

**Figure 1:**
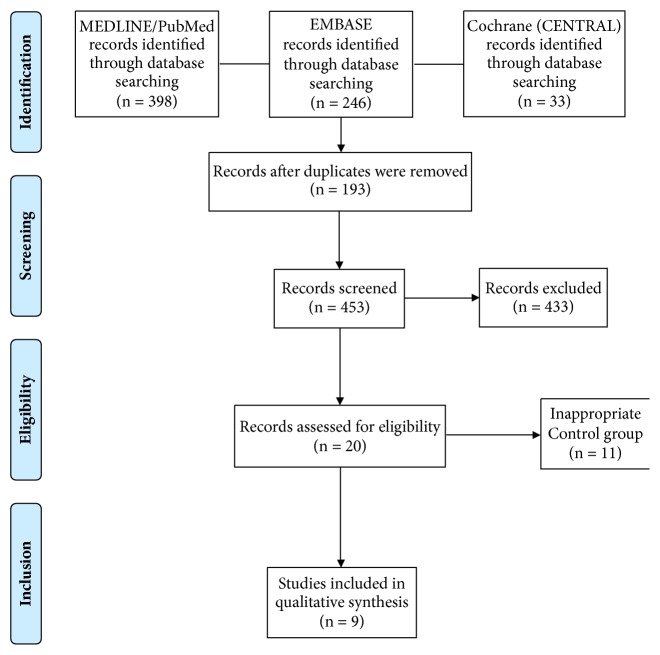
PRISMA flow diagram for the study selection process.

Based on the selection criteria, we included 9 satisfactory studies that were published between 2008 and 2017 in this meta-analysis. The study selection procedure was presented by the PRISMA flow diagram (Figure 1).  Table 1 illustrates the major characteristics of all studies in this meta-analysis. The nine studies are all RCTs (four with a parallel design and five with a split-mouth design) [24, 26–33]. A total of 259 patients with periodontal intrabony defects were treated. The follow-up period ranged from 6 to 12 months.

DFDBA+PRP/PRF/EMD/AM was taken as the test group, with DFDBA alone as the control group. In terms of smoking, except 7 smoker volunteers (2.70%, 7/259) that were recruited in one study [32], all subjects in the other eight studies were nonsmokers. None of the studies acquired the highest score in the quality analysis. Allocation concealment was not reported by any of the included studies. Thus, it was regarded as an uncertain risk bias. The risk of bias was estimated to be low for 1 study and moderate for 8 studies (Table 2).

### 3.2. Meta-Analysis

**Figure 2:**
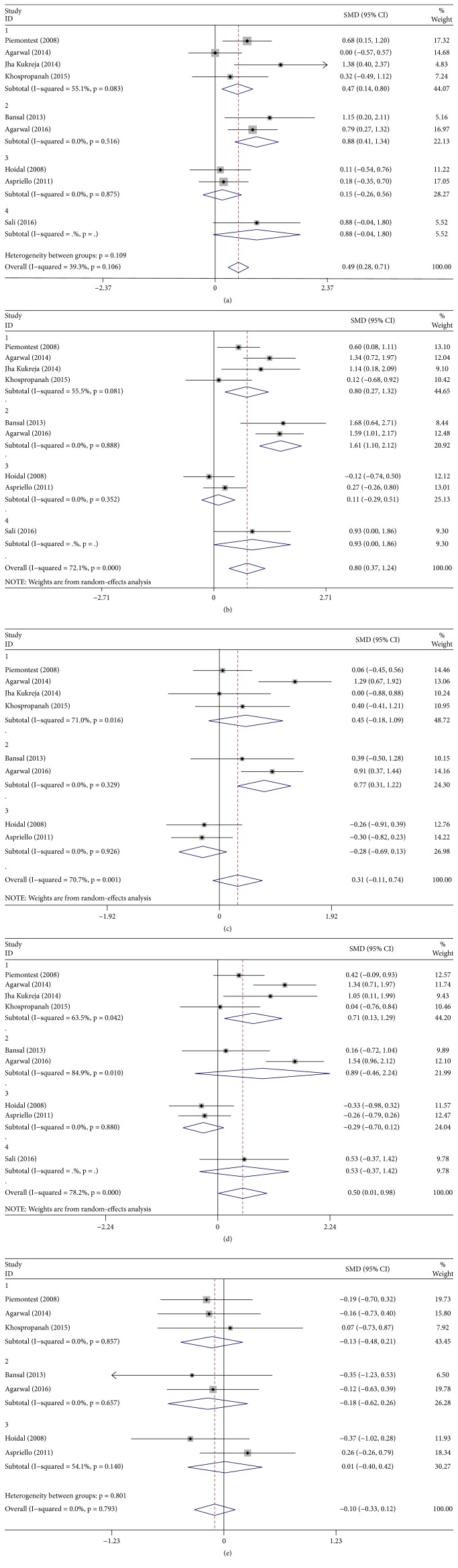
Forest plot for (a) PD gain, (b) CAL, (c) REC, (d) bone fill, and (e) bone resorption. Subgroup 1, PRP+DFDBA versus DFDBA; subgroup 2, PRF+DFDBA versus DFDBA; subgroup 3, EMD+DFDBA versus DFDBA; subgroup 4, AM+DFDBA versus DFDBA.

The results of this meta-analysis were summarized with five forest plots (Figure 2). In the present study, a fixed-effects model was used for evaluating the PD reduction (Figure 2(a)) because of the low heterogeneity that was found among the subgroups (*I*^2^ = 39.3%). The subgroups of PRP and PRF showed statistically significant differences compared with DFDBA alone, with an MD of 0.47 (95% CI = 0.14 to 0.80) and an MD of 0.88 (95% CI = 0.41 to 1.34). PRF subgroup showed better reduction of PD compared to PRP subgroup. In contrast, subgroups of EMD and AM showed no significant PD reduction.

For CAL gain (Figure 2(b)), the random-effects model was employed because of the high heterogeneity (*I*^2^ = 72.1%). The subgroups of PRP, PRF, and AM all showed statistically significant differences compared with DFDBA alone, with an MD of 0.80 (95% CI = 0.27 to 1.32), an MD of 1.61 (95% CI = 1.10 to 2.12), and an MD of 0.80 (95% CI = 0.37 to 1.24), respectively. PRF subgroup showed best gain of CAL compared with the subgroups of PRP and AM. EMD subgroup failed to show any significant difference.

For RecRed (Figure 2(c)), the random-effects model was used on account of the high heterogeneity (*I*^2^ = 70.7%). Only PRF subgroup showed a statistically significant difference compared with DFDBA alone, with an MD of 0.77 (95% CI = 0.31 to 1.22). The subgroups of PRP, EMD, and AM showed no significant differences. For bone fill (Figure 2(d)), the random-effects model was used due to the high heterogeneity (*I*^2^ = 78.2%). Only PRP subgroup showed a statistically significant difference compared with DFDBA alone, with an MD of 0.71 (95% CI = 0.13 to 1.29). The subgroups of PRF, EMD, and AM showed no significant differences. For bone resorption (Figure 2(e)), the fixed-effects model was used because of the low heterogeneity (*I*^2^ = 0%). Nevertheless, all of the subgroups showed no significant difference compared with DFDBA alone.

### 3.3. Sensitivity Analyses

**Figure 3:**
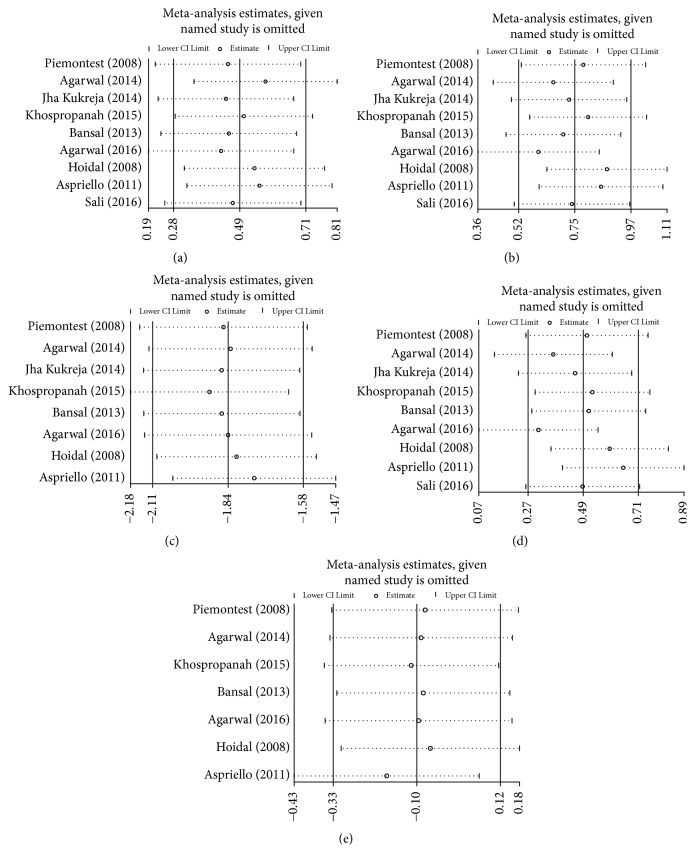
Sensitivity analysis comparing DFDBA+PRP/PRF/EMD/AM versus DFDBA alone. (a) PD reduction; (b) CAL gain; (c) RecRed; (d) bone fill; (e) bone resorption.

Sensitivity analyses were performed by removing one study each time to assess the influence of an individual study on the overall outcomes. The results were stable, indicating that no single study interfered with the overall results significantly (Figure 3).

### 3.4. Publication Bias

**Figure 4:**
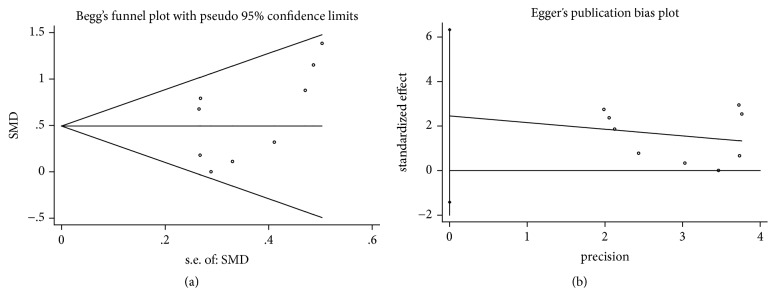
Publication bias of PD reduction of DFDBA+PRP/PRF/EMD/AM versus DFDBA alone. (a) Begg's funnel plot; (b) Egger's publication bias plot.

**Figure 5:**
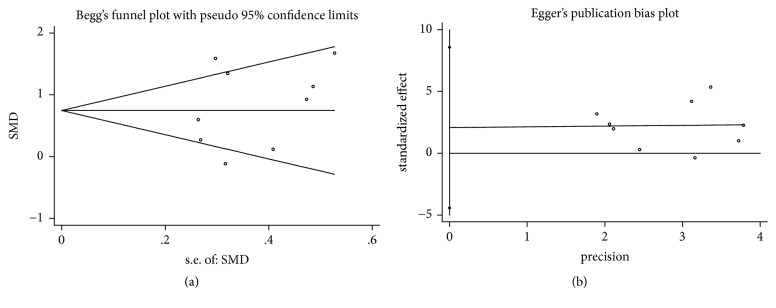
Publication bias of CAL gain of DFDBA+PRP/PRF/EMD/AM versus DFDBA alone. (a) Begg's funnel plot; (b) Egger's publication bias plot.

**Figure 6:**
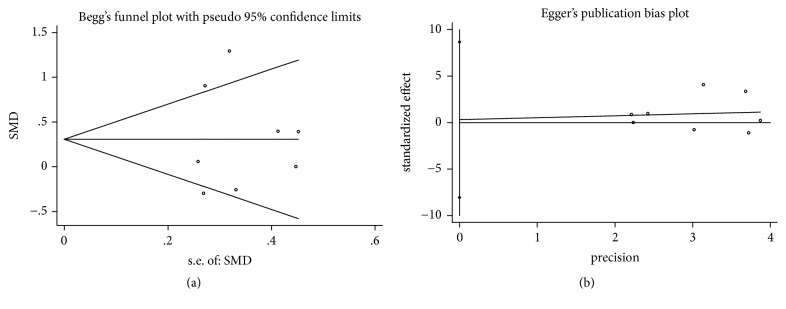
Publication bias of RecRed of DFDBA+PRP/PRF/EMD/AM versus DFDBA alone. (a) Begg's funnel plot; (b) Egger's publication bias plot.

**Figure 7:**
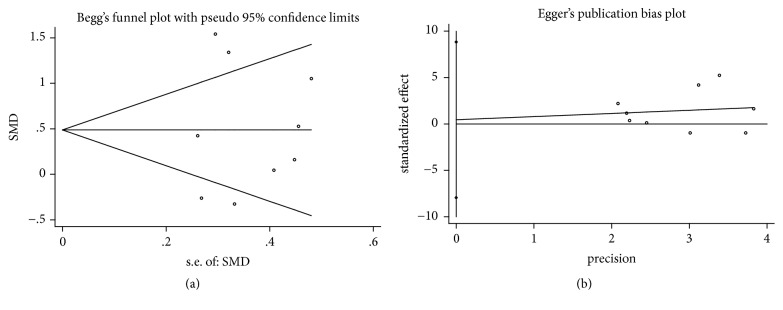
Publication bias of bone fill of DFDBA+PRP/PRF/EMD/AM versus DFDBA alone. (a) Begg's funnel plot; (b) Egger's publication bias plot.

**Figure 8:**
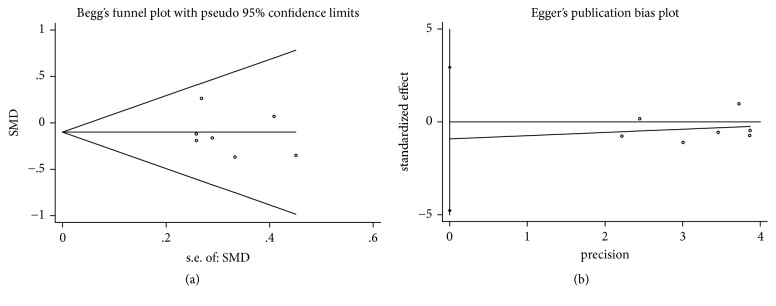
Publication bias of bone resorption of DFDBA+PRP/PRF/EMD/AM versus DFDBA alone. (a) Begg's funnel plot; (b) Egger's publication bias plot.

In the process of Begg's and Egger's test, no publication bias was detected in all assessments (Figures 4, 5, 6, 7, and 8).

## 4. Discussion

Although many studies have claimed that addition of various biomaterials can enhance the regenerative outcomes compared with using scaffold materials alone in periodontal defect, quite a few studies failed to show any significant clinical improvements after combination therapies were used [32, 34–42]. Thus, research on whether bioactive materials will predictably bring to the scaffold materials additional regenerative effectiveness is still far from being conclusive. This systematic review and meta-analysis attempted to evaluate and compare the effectiveness of four different types of biomaterials (EMD, PRP, PRF, and AM) in conjunction with DFDBA by analyzing the changes of clinical and radiographic parameters of PD reduction, CAL gain, RecRed, bone fill, and bone resorption in patients with periodontal intrabony defects, solely relying on the RCTs. DFDBA was here chosen as the scaffold to fill the periodontal defect because it is the most commonly used bone replacement graft and is approved by the FDA as a truly osteoinductive material [43]. The evaluation period of >=6 months was selected because of the fact that this is the usual time frame used in most clinical studies to assess the outcomes of reconstructive periodontal surgery.

The findings from the meta-analysis have demonstrated that both PRP and PRF positively influenced CAL gain and PD reduction, whereas the use of PRF led to significantly better CAL gain (with an MD of 1.61 (95% CI = 1.10 to 2.12) versus an MD of 0.80 (95% CI = 0.27 to 1.32)) and PD reduction (with an MD of 0.88 (95% CI = 0.41 to 1.34) versus an MD of 0.47 (95% CI = 0.14 to 0.80)) than that of PRP. Moreover, for RecRed, only PRF showed a statistically significant difference compared with DFDBA alone, with an MD of 0.77 (95% CI = 0.31 to 1.22). Although PRP and PRF are both blood extracts in which platelets are enriched and various growth factors are highly concentrated, they have different biological performances and mechanical properties owing to their different preparation approaches [13]. For PRP preparation, whole blood with anticoagulants needs to be centrifuged twice; after the first centrifugation, the platelet-poor plasma in the upper layer, the “yellow” part in the middle, and a few red blood cells are carefully collected (pipetting) and centrifuged again in order to obtain the intermediate layer, that is, PRP, which is liquid [13, 44, 45]. When they are used in gel form, bovine thrombin and calcium chloride are added to activate the formation of fibrin network, though thin and noncondensed. Compared with PRP, the preparation of PRF is much easier because it does not need additional anticoagulants and chemical activators. After centrifugation, PRF, as a fibrin clot, is obtained in the middle of the tube, which is ready to be used [44]. PRF, rich in fibrin, platelets, leucocytes, monocytes, and stem cells, has been proven to have advantages in regeneration and tissue healing. In contrast to PRP, PRF contains a higher concentration of growth factors and matrix proteins, which are released more slowly and constantly due to the three-dimensional architecture of the adhesive glycoproteins in the fibrin [45]. Moreover, PRF is endowed antimicrobial and anti-inflammatory properties by the concentrated leucocytes trapped in the fibrin mesh [44, 46, 47]. Another superiority of PRF over PRP lies in the mechanical strength of the condensed and strong fibrin-rich membrane matrix of PRF, which is more suitable for manipulation and space maintenance [13]. In addition to being placed into the defect, compressed PRF can be used to cover the defect similar to a guided tissue regeneration (GTR) membrane, serving as a degradable scaffold that facilitates the development of vascularization and guides epithelial cell migration to its surface [47]. In Li et al.'s study, it was found that subcutaneous PRF was partially replaced with collagen fibers 2 weeks after implantation [48]. Therefore, it is understandably easy to interpret the better soft tissue healing of PRF than that of PRP found in this analysis.

However, PRF failed to show any additionally favorable effect on bone fill according to the result of this analysis. Among the four biomaterials investigated, only PRP positively influenced radiographic outcomes of bone regeneration. Arora et al. demonstrated the growth factor differences between PRP and PRF [49]. It was found that significantly higher TGF-ß1, PDGF-AB, and VEGF were released from activated PRP than PRF released. TGF-ß1 stimulates osteoblast precursor cells and promotes bone matrix synthesis. It also regulates PDGF release that plays a crucial role in new bone formation. VEGF potently accelerates early angiogenesis and wound healing, while it enhances bone regeneration at a later time point [49, 50]. So the result of this analysis may affirm the fact that the adjunctive use of PRP together with conventional grafting procedures may be more helpful for the bone repair of periodontal intrabony defects. Nevertheless, quite a lot of studies substantiated a positive impact of PRF on bone healing [34, 35]. The inconsistency of the outcomes of studies may be attributed to the differences in the methodologies employed to obtain the PRP/PRF preparations. Although PRP/PRF has been extensively used for clinical application involving regeneration, there is no clear standard protocol per surgical procedure. For example, the volume of blood drawn for preparation, the amount of PRP/PRF for use, the type of centrifuge, and setting vary from one study to another, which might have an effect on the concentration of growth factors, inflammatory cytokines, or other biomolecular components. So the possibly inconsistent characteristics of PRP/PRF from different studies may limit the data interpretation. Therefore, it is necessary to follow more standardized protocols for the processing and management of PRP/PRF in order to better evaluate and compare their adjunctive effects. On the other hand, the remodeling of hard tissue will consume far more time than 12 months, which is the longest follow-up duration in this study. It was reported that the improved bone fill and linear bone growth continued to improve over 36 months, reaching maximal statistical significant bone fill [51]. Hence, longer follow-up duration should be designed in order to make assessment on bone regeneration more precisely.

EMD has been available as a biologic periodontal regenerative material in the past decades. Its effects have been extensively evaluated and compared with those of open flap debridement (OFD) and other surgical procedures such as bone grafts, GTR, or combined treatments for periodontal intrabony defects [36–39]. A research found that EMD induces angiogenesis of human microvascular cells as well as proliferation and viability at periodontal pocket [52]. However, the role of EMD still remains controversial. The majority of studies indicated that there was almost no added advantage of EMD when used in conjunction with bone substitute materials (e.g., bovine-derived natural bone mineral, nanocrystalline hydroxyapatite, and biphasic calcium phosphate) [12, 32, 40–42]. Consistent with these results, a finding of this current analysis was that no statistical differences were found in clinical outcomes between EMD combined with DFDBA and DFDBA alone. Intini et al.'s in vivo investigation found that EMD had limited ability to induce bone formation; in the other two well-controlled animal studies, EMD was also shown to be nonosteoinductive [53–55]. The insufficient amount of growth factors in commercially available EMD and its unspecific osteoinductive activity may be responsible for the ineffective bone regeneration. Moreover, due to the strict screening criteria, the current analysis only includes two RCTs about EMD. Therefore, more large-scale RCTs are required in order to define the long-term benefits of EMD in combination therapies.

AM, the most internal placental membrane that is adjacent to the fetal tissue, has unique properties in wound healing. The clinical application of AM (fresh) dates back to the early twentieth century, with the initial utilization in burn wounds and skin transplantation [16–18]. Cryopreserved amnion was later used in ophthalmic surgery [56]. Koob et al. evaluated and qualified the growth factors in dehydrated human amnion-chorion membrane products, revealing the quantifiable levels of PDGF-AA and PDGF-BB, TGF-*α* and TGF-*β*1, basic fibroblast growth factor (bFGF), EGF, and granulocyte colony-stimulating factor (G-CSF) [57]. Recently, fresh/cryopreserved amnion, dehydrated amnion laminate, and amnion-chorion membrane have been used in dentistry. AM was proven to be as effective as PRF in the aspect of root recession coverage when combined with coronally advanced flap (CAF) technique [58]. Similarly, the augment in width of keratinized gingiva with AM was found in the treatment of gingival recession, though not as effective as connective tissue graft (CTG) [59, 60]. A clinical study confirmed the effectiveness of AM in the regenerative periapical endodontic surgery combined with HA and PRF to enhance clinical outcomes with decreased postoperative discomfort [61]. Amnion-chorion membranes were also used in alveolar ridge preservation and maxillary sinus membrane repair [62, 63]. A case series demonstrated the positive outcomes of GTR with amnion-chorion membrane as the barrier [23]. Nevertheless, from the results of this analysis, it could be interpreted that AM did not show statistically significant added benefit to the clinical outcomes except for CAL gain in the treatment of intrabony defect. The reasons can be ascribed as follows. Firstly, the growth factors contained in AM may be more favorable for soft tissue healing than for hard tissue healing. Secondly, because AM here was used as a barrier membrane, the bioactive molecules contained may have little effect deep into the intrabony defect. Thirdly, only one study on AM available met the selection criteria and was included in this analysis; so the result was deemed to have a high risk of bias. Finally, there are controversial reports on regeneration property of the AM. As we all know, angiogenesis plays an important role in various regeneration processes. Niknejad et al., however, have found that AM possessed antiangiogenic effect and induced less vessel sprout number and shorter vessel length [64, 65]. Amnion epithelial cells were also reported to induce apoptosis by downregulation of HSP90 (heat shock protein 90) and its client proteins [64]. Overall, the role of AM as an adjunct to repair the intrabony defect still remains questionable. There is a need for further research to better understand its effects in this area.

An important strength of the current analysis was from the study selection. Bias is more likely to exist in nonrandomized studies than in RCTs [66]. RCTs are regarded as the most reliable form of scientific evidence for evaluating the effectiveness of clinical treatments/interventions in the hierarchy of evidence because they reduce spurious causality and bias. So all of the studies included were RCTs in this analysis and systematic review, notwithstanding that this strict inclusion criterion limits the number of studies selected for analysis. In addition, since smoking can reduce immune and fibroblastic function, decrease collagen synthesis, and induce vascular changes, only one study included seven smoker volunteers (2.70%), whose influence may not be adverse [32, 67].

It must be admitted that this analysis has some limitations. First, there is inherent heterogeneity among the studies included. A methodological limitation may have come from the experimental design. Five studies included were designed as split-mouth investigations, while the other four were designed as parallel investigations. Split-mouth experiment is designed to facilitate the comparison of both groups under similar healing conditions by eliminating patient-specific characteristics that might affect the outcomes of conventional and regenerative surgeries, which is relatively superior to parallel experiment. Heterogeneity may also have partially originated from the types of intrabony defects. In this analysis, one-, two-, and three-wall intrabony defects were indiscriminately considered together, whereas the number of intact osseous walls of defects may influence the prognosis of regenerative surgeries. Furthermore, the CAL gain after periodontal regeneration seems to be related to native gingival regenerative capacity [68]. Some other heterogeneities from patient populations, processing methods of biomaterials, surgical techniques, and follow-up durations can also more or less have effects on the results. Additionally, limited data available and the relatively small sample size discounted the authority of this analysis. Hence, more large multicenter RCTs in the future, with standardized protocols to eliminate heterogeneities, would help to make a definitive decision on the option of biological modifiers to enhance the regenerative efficacy in the treatment of periodontal intrabony defects.

## 5. Conclusion

Within the limitation of this analysis, it is indicated that PRF exerts the most significant adjunctive effect on soft tissue healing, while PRP exhibits a unique impact on hard tissue reconstruction in the treatment of periodontal intrabony defect. EMD and AM demonstrated little additional benefit. Therefore, it seems reasonable to suggest that the autologous PRF/PRP could be taken as a preferred adjunct to promote periodontal regeneration due to its proven good biological effects, low costs, and ease of preparation. Nevertheless, standardization of the protocol for the preparation and application of PRF/PRP is needed to obtain an optimal effect in regenerative procedures.

## Figures and Tables

**Table 1:** Characteristics of all studies in meta-analysis.

Authors (year)	Study design, Follow-up	No. of participants (defects), gender, age (mean/range), smoking (no, yes, unclear)	Intrabony defect degree Groups, C: control, T: test
Piemontest et al. (2008)	RCT (parallel) 12 months	60 (60) Gender: female 29, male 31 Mean age: T 58.9 ± 8.6, C 57.4 ± 11.4 Age range: from 47 to 72 Smoking: no	2 or 3 walls IBDs C: DFDBA (*n* = 30) T: DFDBA + PRP (*n* = 30)

Agarwal et al. (2014)	RCT (split-mouth) 12 months	24 (48) Gender: female 10, male 14 Mean age: ? Age range: from 30 to 65 Smoking: no	1 or/and 2 walls IBDs C: DFDBA (*n* = 24) T: DFDBA + PRP (*n* = 24)

Jha Kukreja et al. (2014)	RCT (parallel) 6 months	20 (20) Gender: female 9, male 11 Mean age: ? Age range: from 20 to 47 Smoking: no	At least 1 wall IBD C: DFDBA (*n* = 10) T: DFDBA + PRP (*n* = 10)

Khospropanah et al. (2015)	RCT (split-mouth) 6 months	12 (24) Gender: female 7, male 5 Mean age: 45 Age range: ? Smoking: no	At least 2 walls IBDs C: DFDBA (*n* = 12) T: DFDBA + PRP (*n* = 12)

Bansal et al. (2013)	RCT (split-mouth) 6 months	10 (20) Gender: ? Mean age: ? Age range: ? Smoking: no	IBDS: ? C: DFDBA (*n* = 10) T: DFDBA + PRF (*n* = 10)

Agarwal et al. (2016)	RCT (split-mouth) 12 months	30 (60) Gender: female 14, male 18 Mean age: 52 Age range: ? Smoking: no	2 or/and 3 walls IBDs C: DFDBA (*n* = 30) T: DFDBA + PRF (*n* = 30)

Hoidal et al. (2008)	RCT 6 months	37 (37) Gender: female 13, male 24 Mean age: ? Age range: ? Smoking: yes (7)	1 or/and 2 or/and 3 walls IBDs C: DFDBA (*n* = 20) T: DFDBA + EMD (*n* = 17)

Aspriello et al. (2011)	RCT (parallel) 12 months	56 (56) Gender: female 34, male 22 Mean age: ? Age range: from 48 to 62 Smoking: no	2 or 3 walls IBDs C: DFDBA (*n* = 28) T: DFDBA + EMD (*n* = 28)

Sali et al. (2016)	RCT (split-mouth) 12 months	10 (20) Gender: female 4, male 6 Mean age: ? Age range: from 25 to 45 Smoking: no	IBDs: ? C: DFDBA (*n* = 10) T: DFDBA + AM (*n* = 10)

RCT, randomized controlled trial; C, control; T, test.

**Table 2:** Quality assessment of included studies (*n* = 9).

Authors (year)	RSG	ALC	BOA	ICD	SLR	Risk of bias
Piemontest et al. (2008)	ad	un	Y	N	N	Moderate^a^, 4^b^
Agarwal et al. (2014)	ad	un	Y	N	N	Moderate^a^, 4^b^
Jha Kukreja et al. (2014)	ad	un	un	N	N	Moderate^a^, 3^b^
Khospropanah et al. (2015)	ad	un	Y	N	N	Moderate^a^, 4^b^
Bansal et al. (2013)	ad	un	un	N	N	Moderate^a^, 3^b^
Agarwal et al. (2016)	ad	un	Y	Y	N	Moderate^a^, 3^b^
Hoidal et al. (2008)	ad	un	Y	Y	N	Moderate^a^, 3^b^
Aspriello et al. (2011)	ad	un	Y	N	N	Moderate^a^, 4^b^
Sali et al. (2016)	ad	ad	Y	N	N	Low^a^, 5^b^

RSG, random sequence generation; ALC, allocation concealment; BOA, blinding of outcome assessment; ICD, incomplete outcome data; SLR, selective reporting; ad, adequate; inad, inadequate; un, unknown; Y, yes; N, no. ^a^Three levels of risk of bias: low, all five criteria were met; moderate, 3-4 criteria were met; high, <3 criteria were met. ^b^Number of the assessment categories met.
